# Factors associated with social behaviour for people with Alzheimer’s disease dementia: a video observation study

**DOI:** 10.1002/alz70861_108651

**Published:** 2025-12-23

**Authors:** Jasmine Shaw, Fern Rodgers, Deniz Eda Kavustu, Yuding Wang, Sarah Assaad, Gill Livingston, Andrew Sommerlad

**Affiliations:** ^1^ University College London, London, London UK; ^2^ North London NHS Foundation Trust, London, London UK; ^3^ Institute of Epidemiology and Health | Department of Epidemiology and Public Health University College London, London UK; ^4^ North London NHS Foundation trust, London, London UK

## Abstract

**Background:**

People with Alzheimer’s disease dementia (AD) often experience changes in their social behaviour which is distressing for them and their caregivers. However, little is understood about how social behaviour in conversation is affected by the support provided by or familiarity with their conversation partners. This study aimed to explore quantitatively the influence of support provided by, and familiarity with, conversation partners on the social behaviour of people with mild AD during video‐recorded and rated conversations.

**Method:**

Two independent researchers double‐rated recordings of conversations between 19 participants with mild AD and a friend or family member (familiar conversation partner), and an unfamiliar researcher. We assessed the support provided by conversation partners using the Measure of Support in Conversation‐Dementia (MSC‐D), and the social behaviour of participants with AD dementia using two measures of social behaviour, the Measure of Participation in Conversation‐Dementia (MPC‐D) and Social Observation Inventory (SOI). We used multi‐level modelling to explore adjusted associations of support and familiarity with social behaviour.

**Result:**

Greater support in conversation was associated with more appropriate social behaviour of participants with AD dementia. Every 1‐point increase in MSC‐D score was associated in fully adjusted models with a 0.29 (95% confidence interval 0.14, 0.44) higher MPC‐D score and a 1.59 (0.87, 2.32) higher SOI score. Three MSC‐D subdomains assessing whether the conversation partner ensures the person with dementia understands topics and questions, whether the partner ensures the person with dementia is able to get their message across, and whether the partner ensures that they have correctly received the message from the person with dementia were most strongly associated with social behaviour. However, we found that familiarity with the conversation partner was not associated with the social behaviour of participants with AD dementia.

**Conclusion:**

More support from conversation partners facilitated more appropriate social behaviour for people with AD dementia, suggesting that interventions to improve conversational support from friends and families of people with dementia may be helpful and these should be developed and tested in future research.

